# Supplementary material for: The art of compensation: How hybrid teams solve collective-risk dilemmas
Source: PLoS One. 2024 Feb 9;19(2):e0297213. doi: 10.1371/journal.pone.0297213 (PMC10857581; doi:10.1371/journal.pone.0297213)
Supplement: Attachment — Submitted filename: Response_to_Reviewers.pdf [file pone.0297213.s001.pdf]

# Response to Reviewers

Reviewer #1: In this manuscript, the authors developed an agent-based model where two types of agents play the one-shot collective risk dilemma (CRD). One type is supposed to be a human-like agent. Those agents optimize their strategies for the CRD based on the framework of evolutionary games. The other type is supposed to be AI-like agents. Those agents cooperate in the CRD with probability p. Using this model, the authors find that interesting phenomena shown in Fig. 1B and 2B. When the AI-like agents cooperate with high probability (bottom right area in Fig. 1B), the human-like agents do not cooperate. They free ride on the cooperation of the AI-like agents. On the other hand, when the AI-like agents cooperate with low probability (top left area in in Fig. 1B), the human-like agents actively cooperate to accomplish the goal of the CRD. The authors argued that those two types of agents compensate with each other. Thus, these human-AI hybrid teams works well to prevent the collective risks. Then, the authors considered the situation how the hybrid team works well by substitution or addition of AI-like agents to human-like agents. The model is new and worth investigating from the view points of not only evolutionary games but also human-AI interaction. I suggest two minor comments below. Please take them into account for improving the manuscript more.

We thank the reviewer for the positive assessment of our work. Please find below the answers to your two remaining comments.

1. Obviously, readers would like to know whether real humans and real AI (AI bots) actually compensate with each other when playing the CRD. It can be tested by conducting online experiments. But this is out of the scope of this manuscript because the authors take a model-based approach here. However, it is worth mentioning this point in the Conclusions by using one paragraph. If the authors agree with my suggestion, please do so.

Thank you for your comment, we absolutely agree with the importance of validating our theoretical results with behavioral experiments and have added this remark as a paragraph to our Conclusions. The new paragraph should appear highlighted in the document 'Revised Manuscript with Track Changes' in lines 453-458.

2. It is worth citing the following royal society interface paper because they also simulated human-AI evolutionary games based on a model-based approach.
Small bots, big impact: solving the conundrum of cooperation in optional Prisoner's Dilemma game through simple strategies, Gopal Sharma, Hao Guo, Chen Shen and Jun Tanimoto

Thank you for making us aware of this reference, it is indeed of great interest to our work and we have added it in the Related Work section in line 122 as the new reference [32], as highlighted in the document 'Revised Manuscript with Track Changes'.

Typos:
1. Ref. [32] is double cited on lines 127 and 258.

2. line 280: with the results in 1 --> with the results in 1A

We gladly confirm that the typos found were corrected, and we thank the reviewer again for their time and attention in revising our manuscript and helping us improve our work.
